# Lipid flip-flop and desorption from supported lipid bilayers is independent of curvature

**DOI:** 10.1371/journal.pone.0244460

**Published:** 2020-12-30

**Authors:** Haoyuan Jing, Yanbin Wang, Parth Rakesh Desai, Kumaran S. Ramamurthi, Siddhartha Das

**Affiliations:** 1 Department of Mechanical Engineering, University of Maryland, College Park, Maryland, United States of America; 2 Laboratory of Molecular Biology, National Cancer Institute, National Institutes of Health, Bethesda, Maryland, United States of America; University of Calgary, CANADA

## Abstract

Flip-flop of lipids of the lipid bilayer (LBL) constituting the plasma membrane (PM) plays a crucial role in a myriad of events ranging from cellular signaling and regulation of cell shapes to cell homeostasis, membrane asymmetry, phagocytosis, and cell apoptosis. While extensive research has been conducted to probe the lipid flip flop of planar lipid bilayers (LBLs), less is known regarding lipid flip-flop for highly curved, nanoscopic LBL systems despite the vast importance of membrane curvature in defining the morphology of cells and organelles and in maintaining a variety of cellular functions, enabling trafficking, and recruiting and localizing shape-responsive proteins. In this paper, we conduct molecular dynamics (MD) simulations to study the energetics, structure, and configuration of a lipid molecule undergoing flip-flop and desorption in a highly curved LBL, represented as a nanoparticle-supported lipid bilayer (NPSLBL) system. We compare our findings against those of a planar substrate supported lipid bilayer (PSSLBL). Our MD simulation results reveal that despite the vast differences in the curvature and other curvature-dictated properties (e.g., lipid packing fraction, difference in the number of lipids between inner and outer leaflets, etc.) between the NPSLBL and the PSSLBL, the energetics of lipid flip-flop and lipid desorption as well as the configuration of the lipid molecule undergoing lipid flip-flop are very similar for the NPSLBL and the PSSLBL. In other words, our results establish that the curvature of the LBL plays an insignificant role in lipid flip-flop and desorption.

## Introduction

The trans-bilayer migration of phospholipid molecules in cell membranes [1–3] is vital to the functioning of eukaryotic cells [4]; the migration event impacts, for example, cell signaling [5]; regulation of shape changes of cells, organelles, and vesicles [6,7]; cell homeostasis [4]; maintenance of membrane asymmetry [8,9]; phagocytosis [10]; and apoptosis [11]. When phospholipid migration occurs from the inner or cytoplasmic side of the bilayer to the outer or the exoplasmic side of the bilayer, it is denoted as a “flop”, whereas it is denoted as “flip” when it occurs in the opposite direction [1–3]. Phospholipid translocation within the lipid bilayer (LBL) and the corresponding energy barriers associated with these processes provide vital clues to a myriad of events such as protrusion-mediated membrane-membrane and membrane-protein interactions [12], clustering of ligands and proteins on the plasma membrane (PM) [13,14], pore formation in the PM [15], localizing and activating enzymes on the PM [16], and dictating the activity of lipid anchors [17]. Such energy barriers are inevitably encountered as hydrophilic entities like charged lipid headgroups translocate from one leaflet of the bilayer to the other through the hydrophobic membrane core [1–3]. Over the years, there have been extensive experimental efforts for quantifying these energetic barriers of trans-bilayer lipid migration, the corresponding kinetics of translocation, and the role of the factors like the lipid chain length and head group, membrane packing, and the presence of cholesterol and peptides within the membrane in flip-flop kinetics [18–34]. Similarly, there have been several molecular dynamics (MD) simulation studies providing molecular level insights on the energetics of lipid flip-flop (often quantified by the potential of mean force values obtained as a function of the lipid position within and outside the lipid bilayer or LBL), and detailed structural information on lipid molecules during their course of the flip-flop [35–41]. Marti *et al*. [35,36] did a pioneering work in the coarse-grained (CG) MD simulation of energetics of lipid flip-flop in planar bilayer. However, in these works the lipids were modeled using spring and bead model. Later, Tieleman *et al*. [37] studied the potential of mean force (PMFs) of lipid flip-flop with all- atom (AA) MD simulations: they related the lipid flip-flop with pore formation and provided a framework for others to follow. After that, they also investigated the lipid flip-flop mediated by different chain lengths [38], chain unsaturation [38], and cholesterol concentration [39]. Gurtovenko *et al*. directly observed lipid flip-flop in AA MD simulations by introducing water pores induced by the imbalance of salt concentration across the membrane [40]. Although AA MD simulations can provide great details about lipid flip-flop, it can only be applied to systems of very limited size. On the contrary, CG MD simulations can probe systems of much larger size and are able to provide the correct PMF; however, such CG MD simulation models are unable to accurately capture the water defect and pores, as indicated by Bennett and Tieleman [41].

Interestingly, most of the MD simulation studies on lipid flip-flops have considered a planar LBL. Despite some MD simulations and experiments investigating the role of membrane curvature on lipid diffusion and sorting [42–45] and experimental study [46] directly measuring lipid flip-flops in curved membranes (and confirming that the lipid flip-flop is independent of the membrane curvature for both unsupported and supported LBLs), the energetics of lipid flip-flops and desorption of lipid molecules in highly curved LBLs are largely unknown. This is especially surprising given the influence of the membrane curvature in defining the morphology of cells and organelles, playing important roles in maintaining certain cellular functions [47] and enabling trafficking [47], recruiting, and localizing shape-responsive proteins [48].

In the present study, we employ coarse-grained MD simulations for studying the energetics of flip-flop and desorption of lipid molecules in curved LBLs, represented by nanoparticle-supported lipid bilayers (NPSLBLs). Such NPSLBLs have been extensively used for targeted delivery of drugs and genes [49–53] as well as for characterizing curvature-sensitive molecules [54–56]. The energetics of lipid flip-flop and desorption are quantified by studying the PMF (potential of mean force) of a single lipid molecule as a function of its position within the LBL. In order to pinpoint the exact impact of the curvature on the flip-flop and desorption energetics, we compare our findings with those for a planar-substrate-supported lipid bilayer (PSSLBL). The curvature causes the NPSLBL and the PSSLBL to differ significantly from each other in terms of area per lipid, inner-to-outer leaflet lipid number ratios, etc. Despite that, we find that for both the PSSLBL and the NPSLBL, the PMF profiles are very similar, establishing, most remarkably, very similar energetics of lipid flip-flop and desorption for the cases of PSSLBL and NPSLBL. Therefore, our results establish that the curvature has very little effect on the energetics and mechanisms associated with the lipid dynamics in supported LBL systems. A detailed analysis of the energetics, quantified through the corresponding variation of the PMF, reveal that for both the NPSLBL and the PSSLBL the equilibrium position of the lipid molecule is at either of the inner or the outer leaflet, while the least favorable locations energetically are the hydrophobic core between the two leaflets and the bulk water. In addition, for both the cases, the lipid molecule undergoes a rotation of nearly 180 degrees as it traverses from the inner (outer) to the outer (inner) leaflet (where the lipid molecule is stretched) and occupies a near tangential configuration (in a compressed state) in the hydrophobic core. Finally, we conduct further simulations and establish that the similarity of the PMF profiles (associated with the flip and flop motions and desorption event of the lipid molecules) between the cases of PSSLBL and the NPSLBL are observed for two different materials forming the support (i.e., the NP in the NPSLBL and the planar substrate in the PSSLBL). This result, along with the experimental observation confirming that the lipid flip-flop is independent of the membrane curvature for both unsupported and supported LBLs [46], establishes our study as a generic finding in the context of establishing the role (or the lack of it) of membrane curvature in lipid flip-flop and desorption events.

## Methods

### Self-assembly of the PSSLBL and the NPSLBL

**Fig 1:**
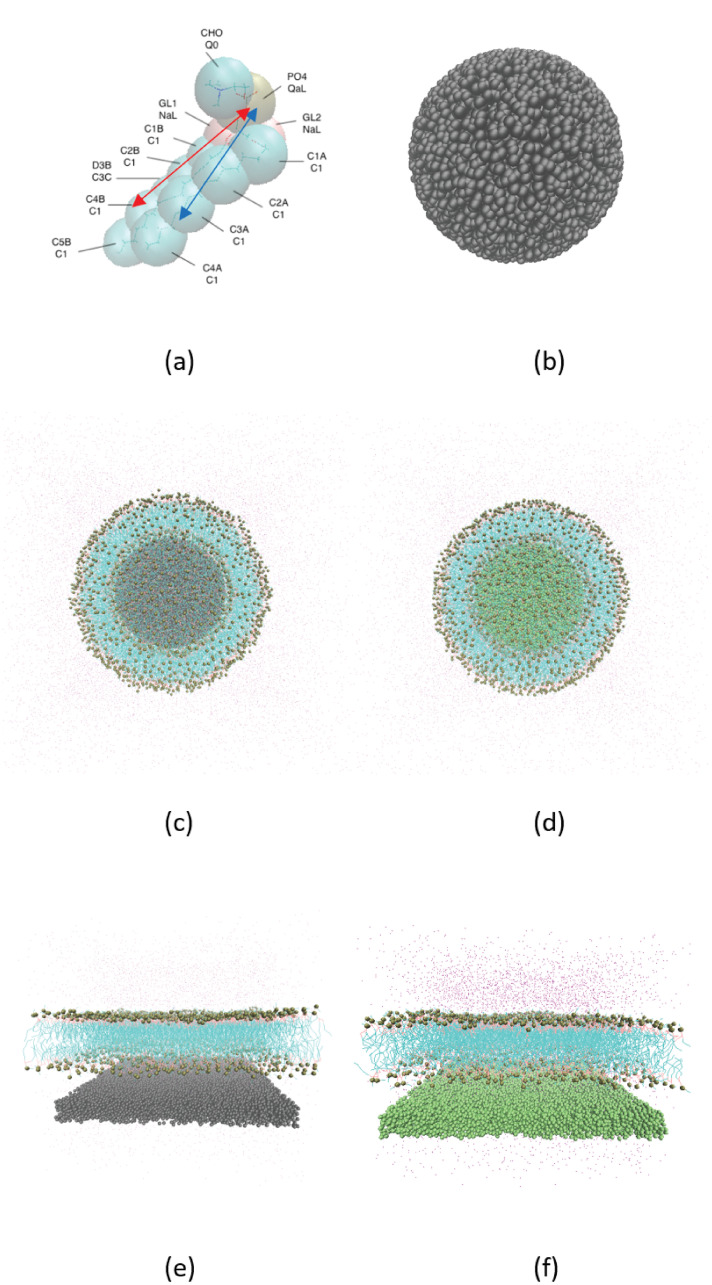
**(a)** Martini model of the POPC lipid molecule, where the lipid molecule is represented by 13 large spheres (beads). Each of the beads is so labelled that their names are identified on the upper row and their types are identified on the bottom row (for example, the name of the “golden” color bead is “PO_4_” and its type is “QaL”). The red double arrow shows the definition of *l*_*B*_, the distances between the PO4 beads and the center of C5B, C4B, and C3C beads while the blue double arrow shows the definition of *l*_*A*_, the distances between the PO4 beads and the center of C2A, C3A, and C4A beads. The figure has been reproduced with permission from Jing, H.; Wang, Y.; Desai, P. R.; Ramamurthi, K.; Das, S. Formation and Properties of Self-Assembled Nanoparticle-Supported Lipid Bilayer Probed Through Molecular Dynamics Simulations. *Langmuir* **2020**, 36, 5524–5533. Copyright (2020) American Chemical Society. (b) Snapshot of the NP. (c) Snapshot of *system A* in its equilibrium configuration. (d) snapshot of *system B* in its equilibrium configuration. (e) Snapshot of *system C* in its equilibrium configuration. (f) Snapshot of *system D* in its equilibrium configuration. Also, please see S8 Fig in the S1 File that confirms the presence of a thin water layer between the NP and the LBL (for the NPSLBL) and the planar support and the LBL (for the PSSLBL).

We used the Martini model [57] for the simulation. The details of the self-assembly process of NPSLBL and PSSLBL have been provided in the supporting materials and also discussed in greater detail in our previous paper [58]. There are two types of NPSLBL systems: system A and system B. System A consists of POPC molecules for lipids and Nda beads for the NP. On the other hand, system B consists of POPC molecules for lipids and P5 beads for the NP. Fig 1(a) and 1(b) respectively show the structure of the lipids and the NP. On the other hand, Fig 1(c) and 1(d) respectively show the systems A and B at their equilibrated configurations. Similarly, we consider two types of PSSLBL systems: system C and system D. System C consists of POPC molecules for lipids and Nda beads for the planar supporting substrate, while *system D* consists of POPC molecules for lipids and P5 beads for the planar supporting substrate. The equilibrated structure of systems C and D are shown in Fig 1(e) and 1(f), respectively. In this context, however, one should be aware of the limitations of the Martini model (in simulating the lipid flip-flop), namely (1) the absence of a dipole in the Martini water model that forbids the reproduction of the hydrophobic effects and (2) the inability of the Martini water model to capture the orientation of the water molecules (this limitation might have critical implication in capturing the substrate-curvature-dependent energetics of lipid flip-flop given the fact that the water will be oriented differently at a curved surface as compared to a flat surface).

In Table 1, we have summarized the key differences in the equilibrium parameters between the NPSLBL and the PSSLBL.

**Table 1:** Differences in the equilibrium configuration properties between the NPSLBL and the PSSLBL.

	*System A*	*System B*	*System C*	*System D*
Area per lipid (*nm*^2^), inner leaflet	0.61	0.62	0.85	0.87
Area per lipid (*nm*^2^), outer leaflet	0.88	0.89	0.82	0.86
Inner-to-outer lipid number ratio	0.64:1	0.65:1	0.95:1	0.98:1

### Potential of mean force calculation

**Fig 2:**
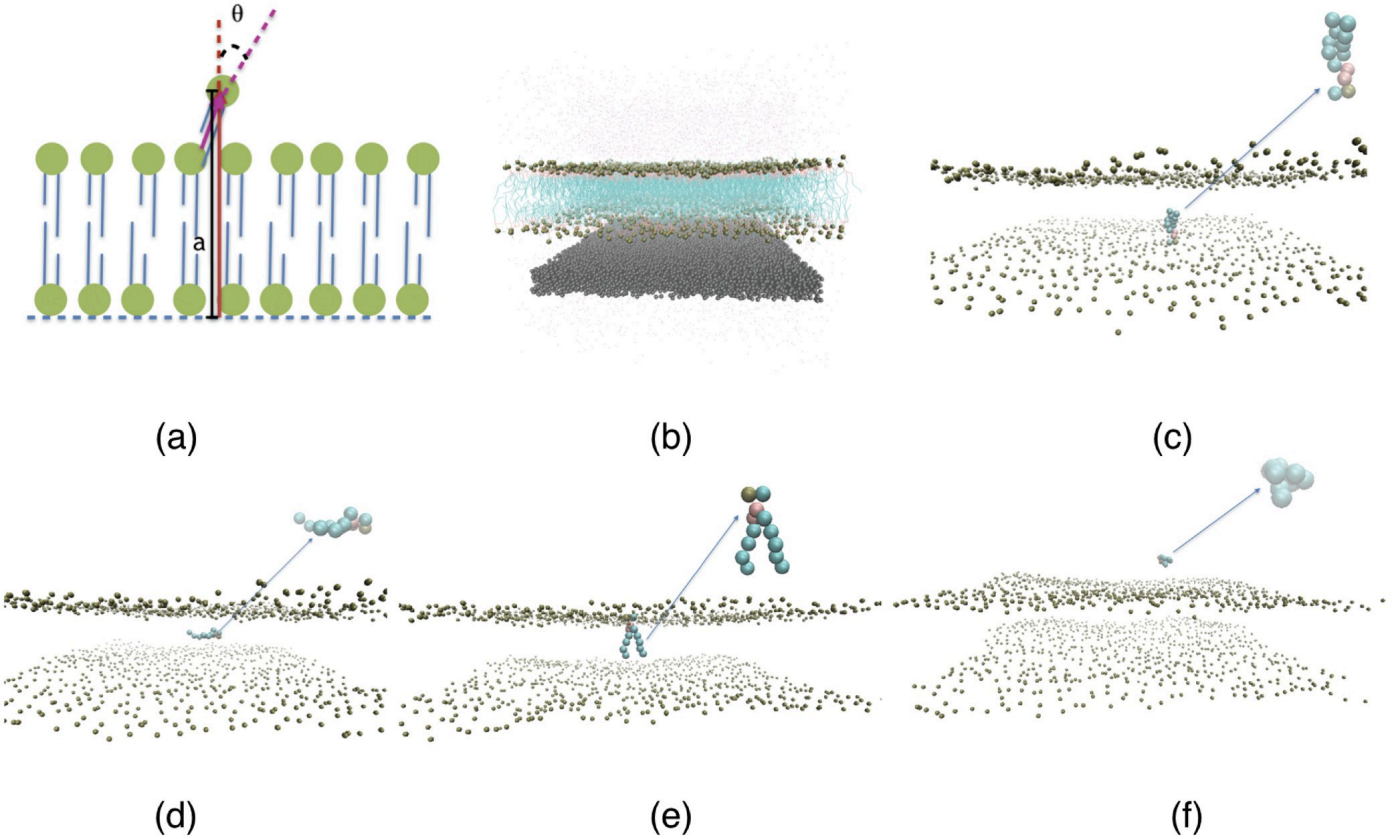
(a) The schematic depiction of the geometry of the PSSLBL (system C). (b) MD simulation snapshot of the PSSLBL; only 1/10^th^ of the total number of water molecules are displayed for a clearer view. (c-f) Snapshots representing the positions (and configurations) of a single lipid molecule (zoomed in the inset showing the corresponding lipid configuration) corresponding to its different locations inside and outside the LBL of the PSSLBL. These different locations are (c) inner leaflet, a = 0 Å; (d) hydrophobic core, a = 22 Å; (e) outer leaflet, a = 44 Å; (f) bulk water, a = 70 Å. For all the cases we use the following color codes: purple for water; dark green for the planar support; light green for the hydrophobic tails of lipids; Bronze for the hydrophilic head of the lipids. For figures (c-f), only the hydrophilic heads of the lipid molecules of the LBL are displayed for a clearer view.

**Fig 3:**
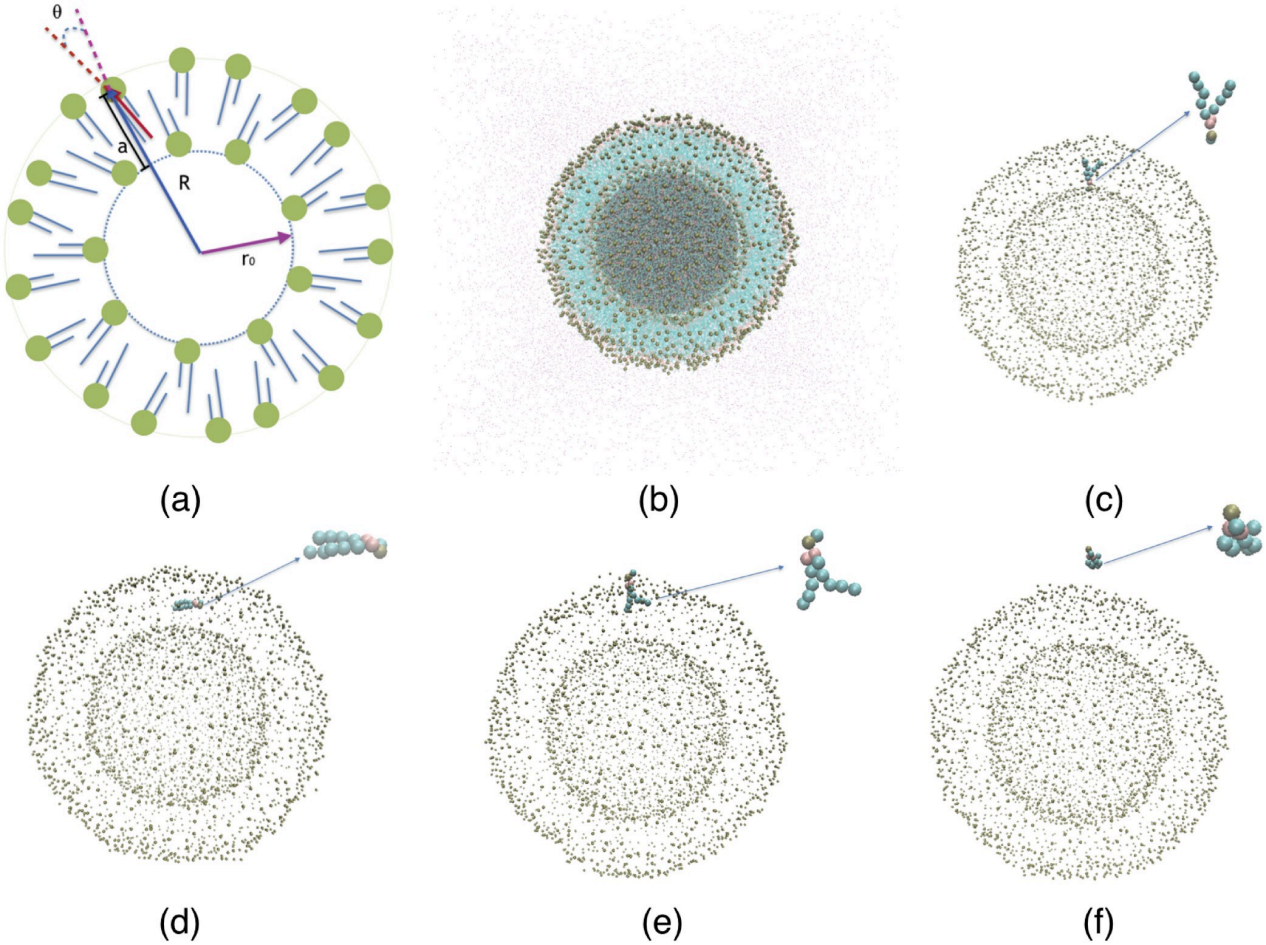
The schematic depiction of the geometry of the NPSLBL (system A). (b) MD simulation snapshot of the NPSLBL; only 1/10^th^ of the total number of water molecules are displayed for a clearer view. (c-f) Snapshots representing the positions (and configurations) of a single lipid molecule (zoomed in the inset showing the corresponding lipid configuration) corresponding to its different locations inside and outside the LBL of the NPSLBL. These different locations are (c) inner leaflet, a = 0 Å; (d) hydrophobic core, a = 22 Å; (e) outer leaflet, a = 44 Å; (f) bulk water, a = 70 Å. For all the cases we use the following color codes: purple for water; dark green for the planar support; light green for the hydrophobic tails of lipids; Bronze for the hydrophilic head of the lipids. For figures (c-f), only the hydrophilic heads of the lipid molecules of the LBL are displayed for a clearer view.

In order to obtain the PMF (quantifying the energetics of lipid flip-flop and desorption) for both the NPSLBL and the PSSLBL, we first identify two lipids (denoted as *star lipids*), with one located at the inner leaflet and the other located at the outer leaflet. Subsequently, we employ the umbrella potential [59] to these two lipids. For the PSSLBL, the reaction coordinate, *α*, is set as the distance along the LBL normal direction between the PO4 beads of the *star lipids* and the inner leaflets of the LBL, as illustrated in Fig 2(a). On the other hand, for the NPSLBL, the reaction coordinate is *α* = *R* − *r*_0_. Here *R* was the distance between the PO4 beads of the *star lipids* and the center of NPSLBL, while *r*_0_ was the radius of the inner leaflets [see Fig 3(a)]. For both cases, *α* ranged from 0 to 70 Å. The *star lipids* were shifted by 2 Å per simulation window, and we considered 35 such simulation windows. The 35 initial structures corresponding to the 35 simulation windows were obtained by pulling the star lipids to their window location via the umbrella potential with a force constant of 2.5 kcal mol^-1^ Å^2^. Each simulation window was equilibrated for 200 ns, followed by a 100 ns production run. The PMFs were constructed from the simulations by using the WHAM [60] program. Figs 2(b) and 3(b) respectively provide the MD simulation based equilibrated structures of the PSSLBL (system C) and the NPSLBL (system A). In Figs 2(c)–2(f) and 3(c)–3(f), we provide the MD simulation snapshots for the different positions of the lipid molecules for quantifying the PMF of the lipid flip-flop and desorption for the PSSLBL and the NPSLBL, respectively.

## Results and discussions

**Fig 4:**
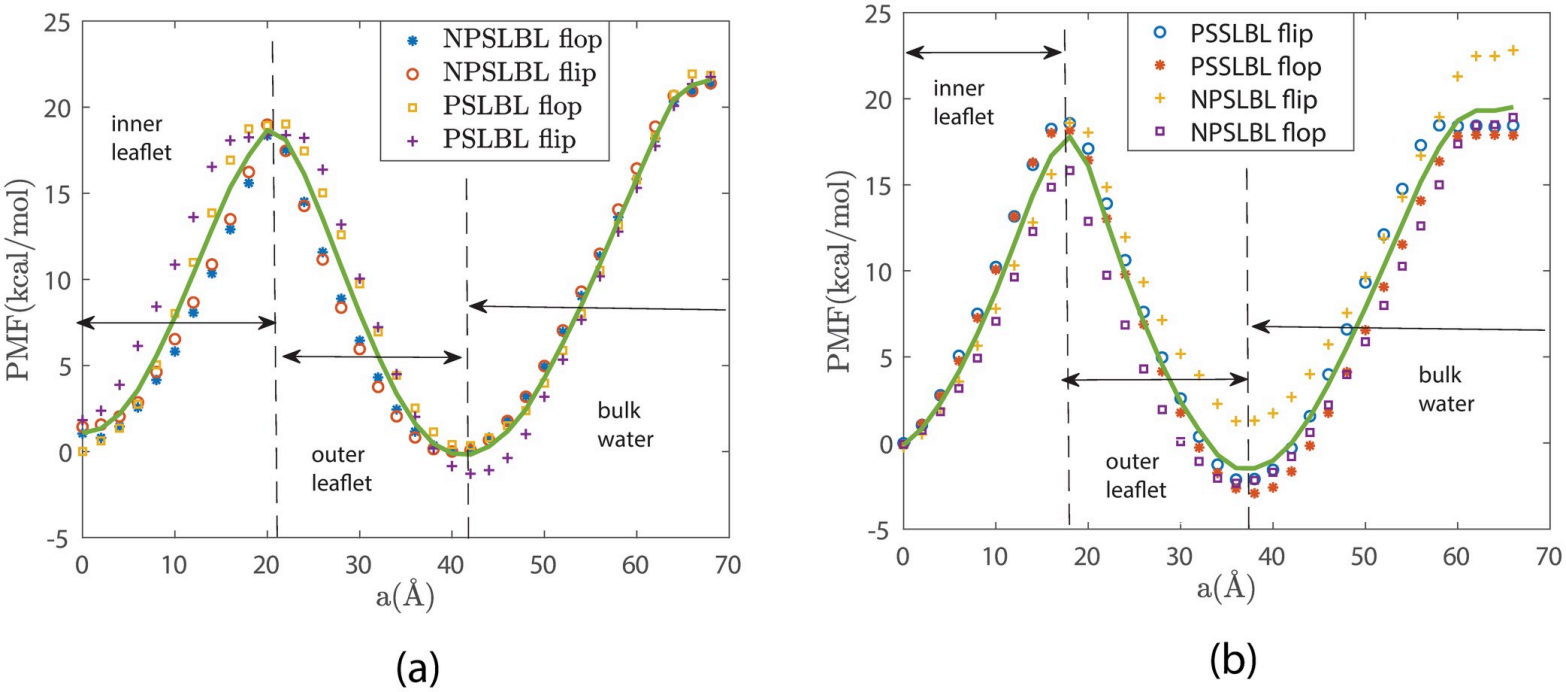
Variation of the PMF [with respect to the reaction coordinate (*a*)] of a single lipid molecule traversing inside the LBL during the flip and the flop motions and outside the LBL during the desorption. Results are shown for systems A (NPSLBL with NP composed of Nda beads) and C (PSSLBL with the planar support composed of Nda beads) in part (a) and for systems B (NPSLBL with NP composed of P5 beads) and D (PSSLBL with the planar support composed of P5 beads) in part (b). The solid lines in parts (a) and (b) are the averages of the simulation data. Also, in both (a) and (b), we identify the locations of the lipid bilayer and the bulk water. Furthermore, a = 0 Å represents the location of the inner leaflet for both the NPSLBL and the PSSLBL.

We first study the energetics of lipid flip-flop and desorption for the NPSLBL and the PSSLBL. We do so by quantifying the corresponding *PMF-vs-a* variation for a lipid molecule for the NPSLBL and the PSSLBL (see Fig 4). The reaction coordinate “*α*” has been defined in Figs 2(a) and 3(a) (as well as in the text above) for the PSSLBL and the NPSLBL, respectively. The results clearly indicate that the PMFs are very similar for the two cases (cases of the NPSLBL and the PSSLBL for either type of support material) for the three different types of motions: (a) “flop” motion (when the lipid molecule moves from the inner to the outer leaflet), (b) “flip” motion (when the lipid molecule moves from the outer to the inner leaflet), and (c) desorption (when the lipid molecule moves from the outer leaflet to the bulk water). These results confirm the most important finding of this study: the energetics of lipid flip-flop and desorption is independent of the curvature in the supported LBL systems. In addition to this overall finding on the energetics, we dissect the PMF curve to understand the position dependent behavior of the lipid molecule during their flip-flop and desorption. Invariably, for both the cases of the NPSLBL and the PSSLBL and for either type of support material, the most stable configurations (or the equilibrium positions) of the lipid molecules are at the inner and the outer leaflets. On the other hand, the energetically most unfavorable location for the lipid molecule is bulk water. The hydrophobic core between the inner and outer leaflets is also energetically unfavorable. As the lipid molecule is pulled away from the inner (outer) lipid leaflet towards the inter-leaflet hydrophobic core during the flop (flip) motion, the lipid molecule experiences energy unfavourability. This energy unfavourability is due to the hydrophilic head of the lipid molecule being forced in a hydrophobic core between the two leaflets. As the lipids are closer to the hydrophobic core of the bilayer, the energy unfavourability attains a maximum at the boundary between the inner and the outer leaflets; subsequently, the energy decreases again to attain another local minimum at the outer (inner) leaflet during the flop (flip) motion. Of course, when the lipids enter into the bulk water from the outer leaflet, the energy unfavourability rises significantly and reaches the peak when the lipid molecule is completely surrounded by water. It is important to emphasize here that the inner and the outer leaflets are interdigitated with each other, i.e., the hydrophobic tails of one leaflet penetrates into the hydrophobic space formed by the tails of the other leaflet. Such an interdigitation is evident from the fact that the thickness of the bilayer is smaller than twice the length of a lipid molecule. Therefore, we define the boundary of the two leaflets as the place where the influence of a leaflet becomes dominant. In this work, the influence is evaluated as the stationary point in the PMF.

From Fig 4(a) we can calculate the free energy barrier for the lipid flop, flip, and desorption (for systems A and C) as 17.9 ± 1.6 kcal/mol, 19.25 ± 1.6 kcal/mol, and 20.77 ± 1.6 kcal/mol, respectively. On the other hand, from Fig 4(b), we obtain free energy barrier for the lipid flop, flip, and desorption (for systems B and D) as 17.59 ± 1.4 kcal/mol, 18.84 ± 1.4 kcal/mol and 21.46 ± 1.4 kcal/mol, respectively. The error is estimated based on the standard deviation. From the figures, it is noticed that the simulation data is more scattered at the outer leaflets than elsewhere. We attribute such a scatter to the constraint of the one-dimensional PMF calculation, which means that the shape of the LBL is not strictly spherical nor planar; under such circumstances, one reaction coordinate cannot fully capture the energetic signature. Experiments have reported an energy barrier of 84–113 kJ/mol (20.1–27.7kcal/mol) for the lipid flip-flop motion for different PSSLBLs (DMPC, DPPC, and DSPC bilayers) at 20.9°C [2]. These experimental results match excellently with our simulation findings. Additionally, recent experiments also point to the fact that the lipid flip-flop events are independent of membrane curvature for both supported and unsupported bilayers [45] further validating our detailed simulation-based observations.

**Fig 5:**
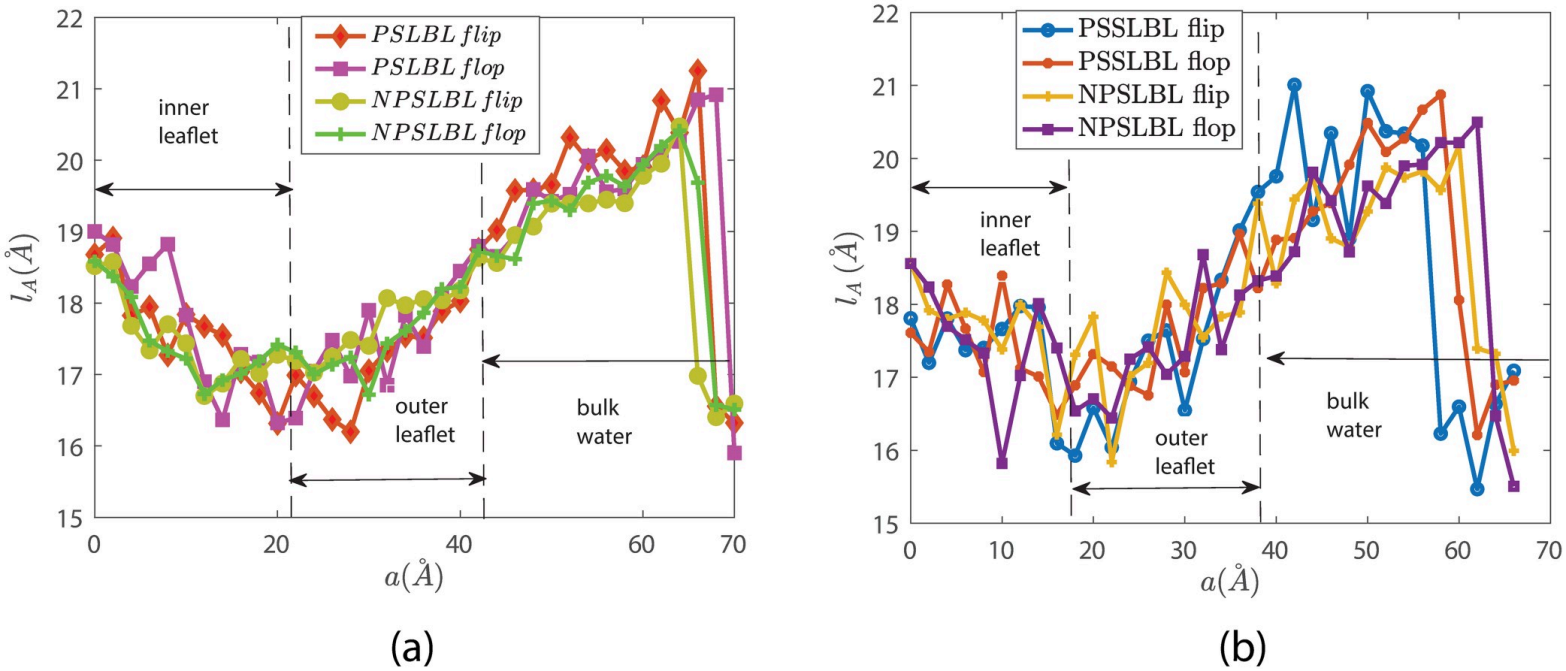
Variation of the tail length *l*_*A*_ for the *star lipid molecules* traversing inside and outside the LBL for the NPSLBL and the PSSLBL. ***l***_***A***_ has been defined in Fig 1(a) and its caption. Results are shown for systems A (NPSLBL with NP composed of Nda beads) and C (PSSLBL with the planar support composed of Nda beads) in part (a) and for systems B (NPSLBL with NP composed of P5 beads) and D (PSSLBL with the planar support composed of P5 beads) in part (b). Also, in both (a) and (b), we identify the locations of the lipid bilayer and the bulk water. Furthermore, a = 0 Å represents the location of the inner leaflet for both the NPSLBL and the PSSLBL.

**Fig 6:**
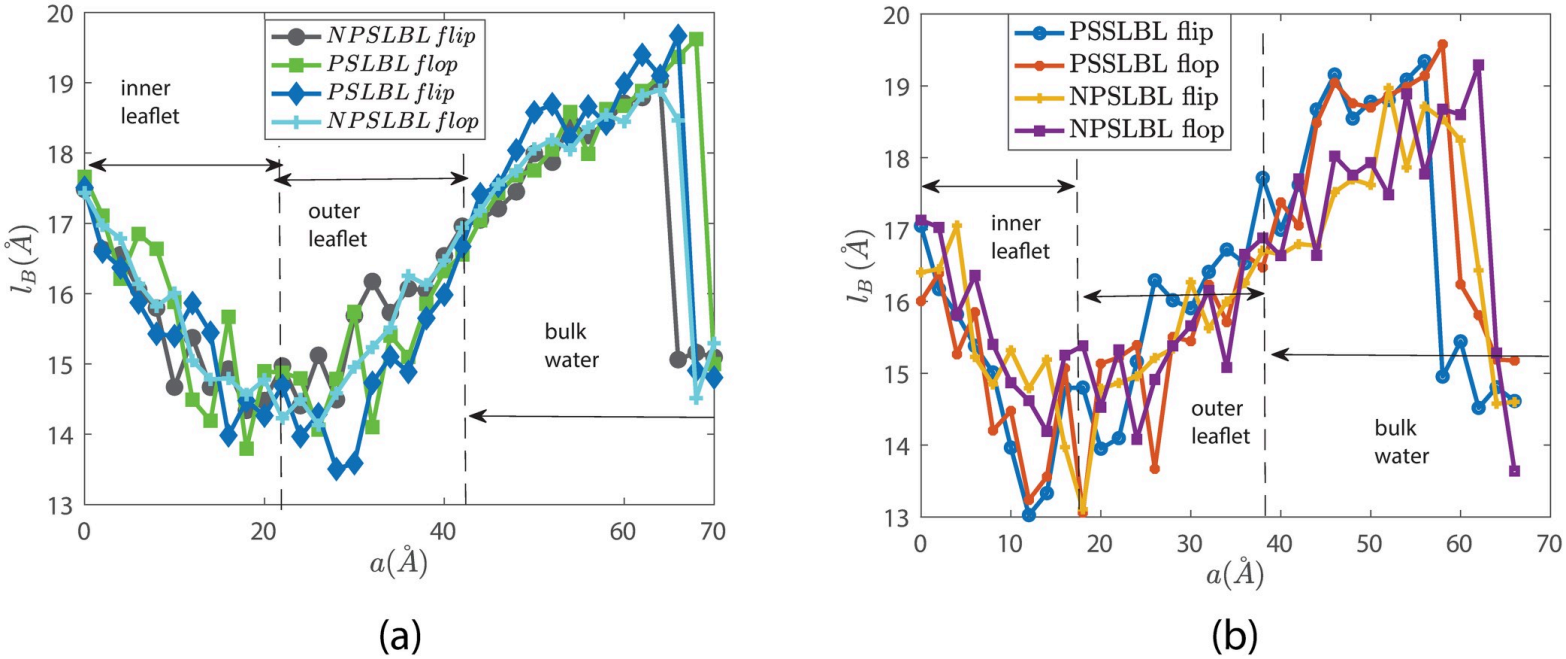
Variation of the tail length *l*_*B*_ for the *star lipid molecules* traversing inside and outside the LBL for the NPSLBL and the PSSLBL. ***l***_***B***_ has been defined in Fig 1(a) and its caption. Results are shown for systems A (NPSLBL with NP composed of Nda beads) and C (PSSLBL with the planar support composed of Nda beads) in part (a) and for systems B (NPSLBL with NP composed of P5 beads) and D (PSSLBL with the planar support composed of P5 beads) in part (b). Also, in both (a) and (b), we identify the locations of the lipid bilayer and the bulk water. Furthermore, a = 0 Å represents the location of the inner leaflet for both the NPSLBL and the PSSLBL.

Figs 5 and 6 show the lengths *l*_*A*_ and *l*_*B*_ of the two hydrophobic tails as the lipids move inside and outside the LBL for the cases of NPSLBL and the PSSLBL. *l*_*A*_ and *l*_*B*_, defined in Fig 1(a) and its caption, are the distances between the PO4 beads and the center of the last three carbon beads of the tails A and B, respectively. Like the PMF variation, the variation for the tail length is similar for the cases of the NPSLBL and the PSSLBL for either type of support material. Both the tails for either of the two cases (NPSLBL or PSSLBL) for either type of support material get compressed as the lipid molecule moves from the inner (outer) leaflet to the hydrophobic core during the flop (flip) motion. This stems from the tendency of the hydrophilic heads to avoid the hydrophobic membrane core. On the other hand, both the tails for either of the two cases (NPSLBL or PSSLBL) for either type of support material get stretched as the lipid molecule moves from the outer leaflet surface into the bulk water, stemming from the tendency of the hydrophobic tails of the lipid molecules to remain localized in the outer leaflet and avoid any contact with water. Finally, when the lipid molecule is entirely in the bulk water (i.e., the lipid molecule has undergone desorption from the LBL), the molecule attains a coil-like shape to minimize its surface area: therefore, the lengths *l*_*A*_ and *l*_*B*_ significantly decrease in the bulk water for both the cases of NPSLBL and the PSSLBL.

**Fig 7:**
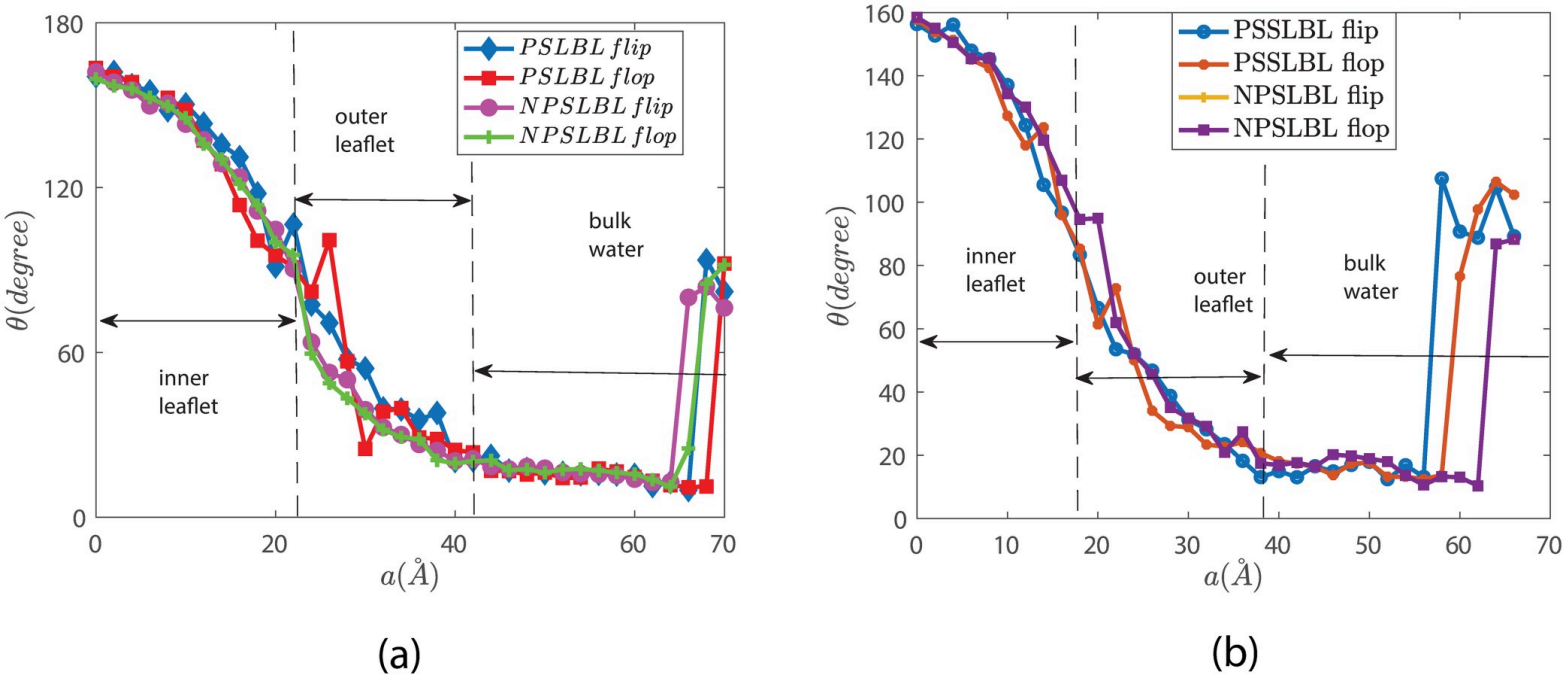
Variation of θ [see Figs 2(a) and 3(a) for definition] for the *star lipid molecule* as it traverses the LBL for both the PSSLBL and the NPSLBL. Results are shown for systems A (NPSLBL with NP composed of Nda beads) and C (PSSLBL with the planar support composed of Nda beads) in part (a) and for systems B (NPSLBL with NP composed of P5 beads) and D (PSSLBL with the planar support composed of P5 beads) in part (b). Also, in both (a) and (b), we identify the locations of the lipid bilayer and the bulk water. Furthermore, a = 0 Å represents the location of the inner leaflet for both the NPSLBL and the PSSLBL.

Finally, Fig 7 shows the orientation of the *star lipid* molecules (defined earlier) as a function of the reaction coordinate *a*. Figs 2(a) and 3(a) provide the definition of *θ* for the PSSLBL and the NPSLBL, respectively. For either type of support material, for both the PSSLBL and the NPSLBL, the *star lipids* are (i) anti-parallel to the membrane normal [also defined in Figs 2(a) and 3(a)] at the inner leaflet (as a consequence, *θ* is nearly 180 degree), (ii) become perpendicular to the membrane normal when they approach the hydrophobic core (as a consequence, *θ* is close to 90 degree), and (iii) become parallel to the membrane normal at the outer leaflet (as a consequence, *θ* is close to 0 degree). The *star lipids* retain this orientation (at the outer leaflet) until they fully merge into the bulk water where they become coil-like, and the definition of the orientation angle become meaningless.

## Conclusions

In this paper, we study the role of the curvature on the energetics of lipid flip-flop and desorption on supported LBLs. Considering NPSLBL and PSSLBL as respective examples of supported curved and non-curved LBLs, our findings establish a highly intriguing finding: the energetics of lipid flip-flop and desorption are independent of curvature. We conduct simulations for two different types of support materials (constituting the NP for the NPSLBL and the planar support for the PSSLBL) and observe that the energetics of lipid flip-flop and desorption remain independent of the curvature for either type of support material. This is most remarkable, given the significant variation in the number distribution as well as area per unit lipid (in the two leaflets) between the cases of NPSLBL and the PSSLBL (see Table 1). The findings also raise the possibility that the lipid flip-flop events might be energetically similar even for curved and non-curved unsupported LBLs (e.g., planar unsupported LBL and vesicles of wide ranges of radii or wide ranges of curvatures). In fact, recent experiments suggest that lipid flop-flop events are independent of the bilayer curvature for both supported and unsupported bilayers [45]. Such experiments, along with our simulations, help to establish the generality of the phenomenon of curvature independence of the energetics of the lipid translocation events such as flop and flip (within the bilayer) and desorption (from the bilayer to the bulk).

## Supporting information

S1 Fig (PDF)

S1 File (PDF)
