# Supplementary material for: Lipid flip-flop and desorption from supported lipid bilayers is independent of curvature
Source: PLoS One. 2020 Dec 30;15(12):e0244460. doi: 10.1371/journal.pone.0244460 (PMC7773258; doi:10.1371/journal.pone.0244460)
Supplement: S1 Fig — (PDF) [file pone.0244460.s001.pdf]

Copyright Clearance Center RightsLink®

Home | Help | Email Support | Sign in | Create Account

Formation and Properties of a Self-Assembled Nanoparticle-Supported Lipid Bilayer Probed through Molecular Dynamics Simulations

Author: Haoyuan Jing, Yanbin Wang, Parth Rakesh Desai, et al

Publication: Langmuir

Publisher: American Chemical Society

Date: May 1, 2020

Copyright © 2020, American Chemical Society

PERMISSION/LICENSE IS GRANTED FOR YOUR ORDER AT NO CHARGE

This type of permission/license, instead of the standard Terms & Conditions, is sent to you because no fee is being charged for your order. Please note the following:

- Permission is granted for your request in both print and electronic formats, and translations.
- If figures and/or tables were requested, they may be adapted or used in part.
- Please print this page for your records and send a copy of it to your publisher/graduate school.
- Appropriate credit for the requested material should be given as follows: "Reprinted (adapted) with permission from (COMPLETE REFERENCE CITATION). Copyright (YEAR) American Chemical Society." Insert appropriate information in place of the capitalized words.
- One-time permission is granted only for the use specified in your request. No additional uses are granted (such as derivative works or other editions). For any other uses, please submit a new request.

If credit is given to another source for the material you requested, permission must be obtained from that source.

BACK CLOSE WINDOW

© 2020 Copyright - All Rights Reserved | Copyright Clearance Center, Inc. | Privacy statement | Terms and Conditions
Comments? We would like to hear from you. E-mail us at customercare@copyright.com
