# Supplementary material for: Lipid flip-flop and desorption from supported lipid bilayers is independent of curvature
Source: PLoS One. 2020 Dec 30;15(12):e0244460. doi: 10.1371/journal.pone.0244460 (PMC7773258; doi:10.1371/journal.pone.0244460)
Supplement: S1 File — (PDF) [file pone.0244460.s002.pdf]

Lipid Flop-flop and Desorption from Supported Lipid Bilayers is Independent of Curvature: Supplementary Material

Haoyuan Jing,[1,2] Yanbin Wang,[1] Kumaran Ramamurthi,[2] and Siddhartha Das[*1]

[1]Department of Mechanical Engineering, University of Maryland, College Park, MD 20742

[2]Laboratory of Molecular Biology, National Cancer Institute, National Institutes of Health

Bethesda, MD 20892, USA

[1]sidd@umd.edu

S1. MD simulations for the self-assembly of the NPSLBL

In our simulations, the POPC molecule is represented by the Martini model,[1] as shown in Fig. 1(a). For the NPSLBL, the NP, as shown in Fig. 1(b), is comprised of Nda beads that were randomly distributed in a spherical shell with an inner radius 5.5 nm and an outer radius 7 nm. There are 3269 POPC molecules randomly distributed around the NP. We name this system as *system A*. The self-assembly of *system A* is shown in Figure S1. In addition, we run a new set of simulation by replacing the Nda beads in *system A* with P5 beads, which is more hydrophilic[1], while the other settings, including the number and type of lipids, simulation parameters, and so forth, are kept the same as *system A.* The new system is called *system B.* The self-assembly of system B is shown in Figure S2.

Initially, the lipid molecules assemble into multiple small clusters [Fig. S1(b, c) and Fig. S2(b, c)]. Subsequently, these clusters localize and assemble near the surface of the NP, forming a LBL that encapsulate the NP [Fig. S1(d) and Fig. S2(d)]. However, the LBL is not sealed, with a few small clusters still undergoing merging inside the water [Fig. S1(e) and Fig. S2(e)]. The temperature of the system is subsequnetly increased from 310K to 340K at the MD simulation step corresponding to the 2 μs [Fig. S1(f) and Fig. S2(f)] to accelerate the simulation. As a result, the LBL gradually got sealed, and the distributed clusters present in water got assembled with the LBL [Fig. S1(g) and Fig. S2(g)]. Consequently, we obtain a perfectly sealed NP-supported LBL, which is equivalent to the NPSLBL [see Fig. 1(h) and Fig. S2(h)]. We equilibrate these NPSLBL systems by running the simulations further for another 2 μs [results are shown in Fig. S1(i) and Fig. S2(i)]. Finally, we decrease the temperature to 310K and equilibrate the system for another 2 μs [results are shown in Fig. S1(j) and Fig. S2(j)]. At equilibrium, there are 1274 lipids at the inner leaflet and 1994 lipids at the outer leaflet of the NPSLBL for *system A*. On the other hand,

there are 1287 POPC molecules located at the inner leaflet of the bilayer, and 1981 POPC beads located at the outer leaflet for *system B*. More details of this self-assembly equilibration of the NPSLBL have been provided in our recent article.[2]

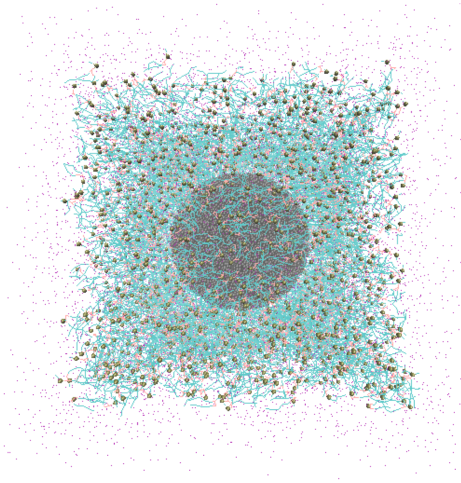

(a) 0 *ps*

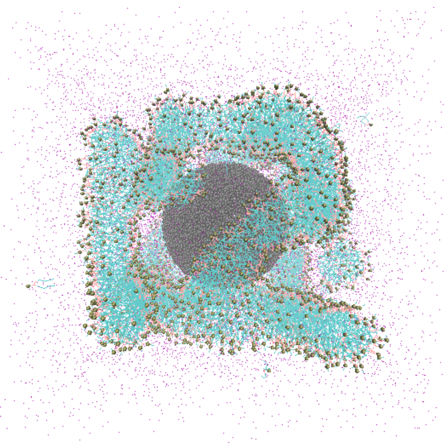

(b) 80 *ps*

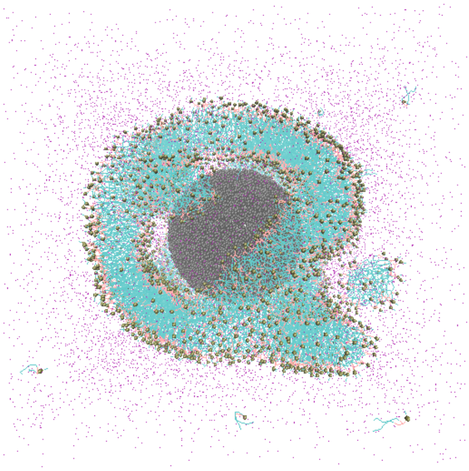

(c) 2 *ns*

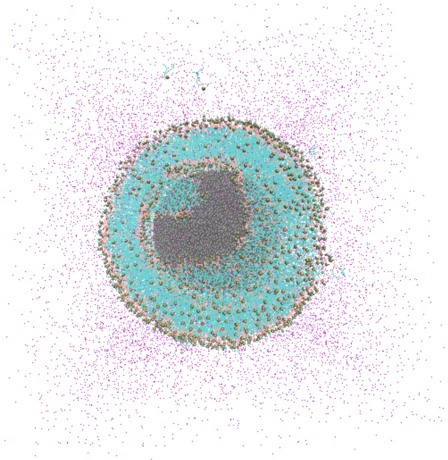

(d) 4 *ns*

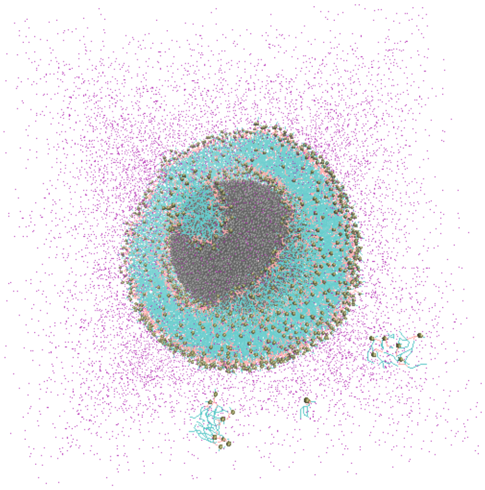

(e) 8 *ns*

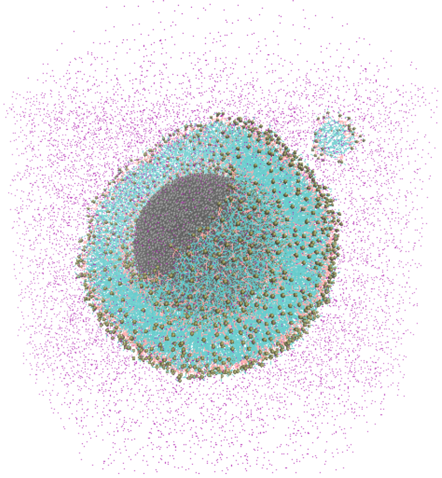

(f) 2 *μs*

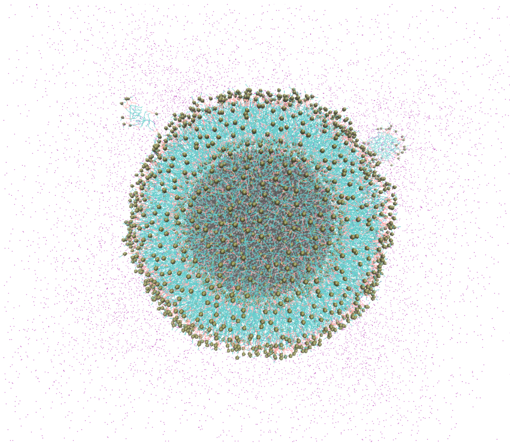

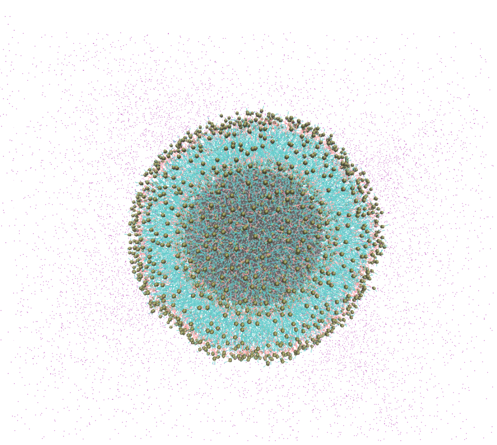

(g) 2.2 μs (h) 2.3 μs

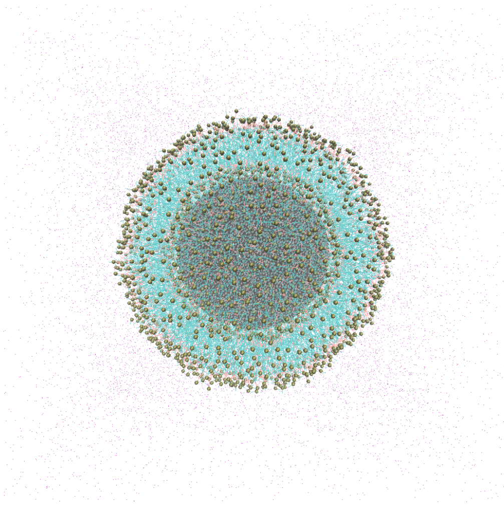

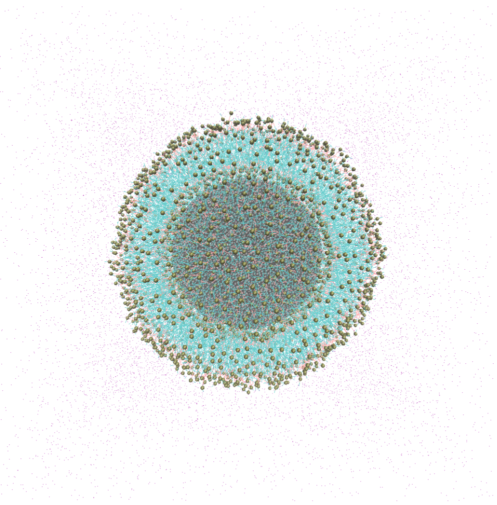

(i) 4.3 μs (j) 6.3 μs

Figure S1: MD simulation snapshots capturing the self-assembly driven equilibration of the NPSLBL system (system A or the system where the Nda beads constitute the NP of the NPSLBL and the lipids are the POPC molecules). The snapshot showing the initial configuration is provided in part (a) while the snapshots quantifying the progress of the simulation are shown in parts (b-h). Also, the snapshots from (a-e) represent the simulations conducted at 310 K, while those from (f-h) represent the simulations conducted at 340 K. In (b) to (h), only 1/10th water are displayed for a clearer view. Below each snapshot, we provide the corresponding value of the time at which the snapshot has been taken. In the different subfigures, the following color codes

have been used: black: NP; light green: hydrophobic tails of POPC molecules; bronze: hydrophilic heads of POPC molecules; purple: water. Part of the figure was reproduced from our previous paper.[2]

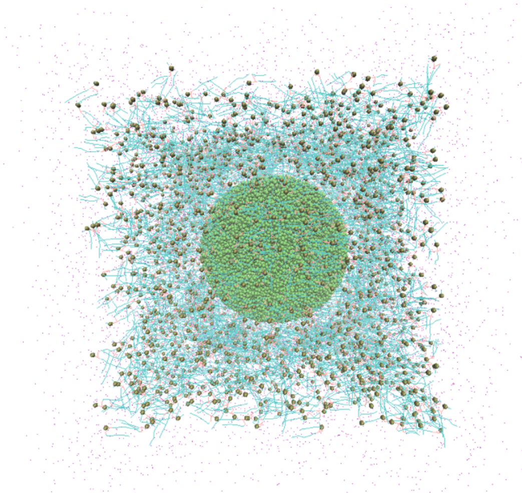

(a) 0 *ps*

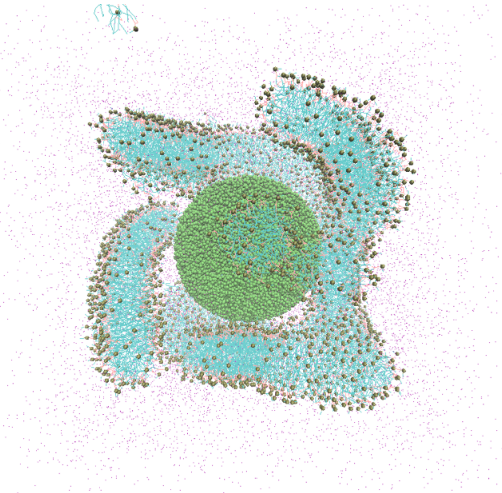

(b) 80 *ps*

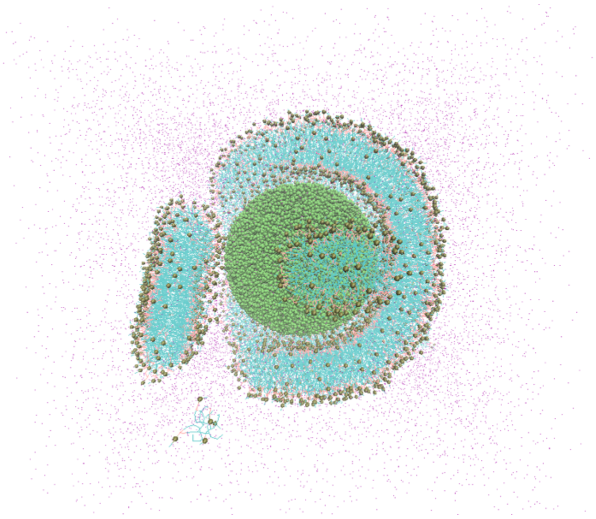

(c) 2 *ns*

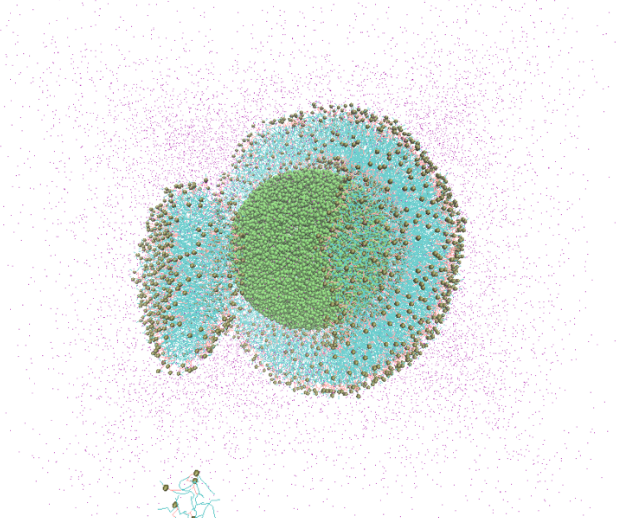

(d) 4 *ns*

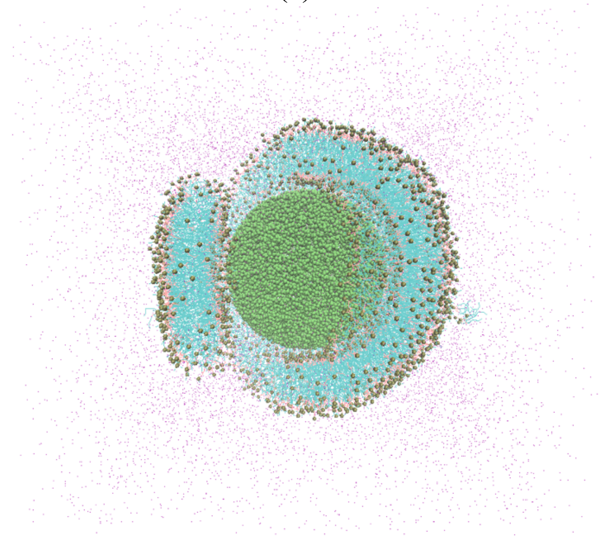

(e) 8 *ns*

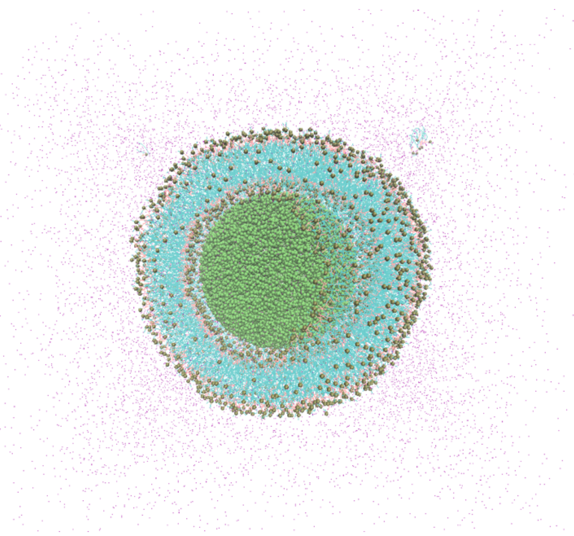

(f) 2 *μs*

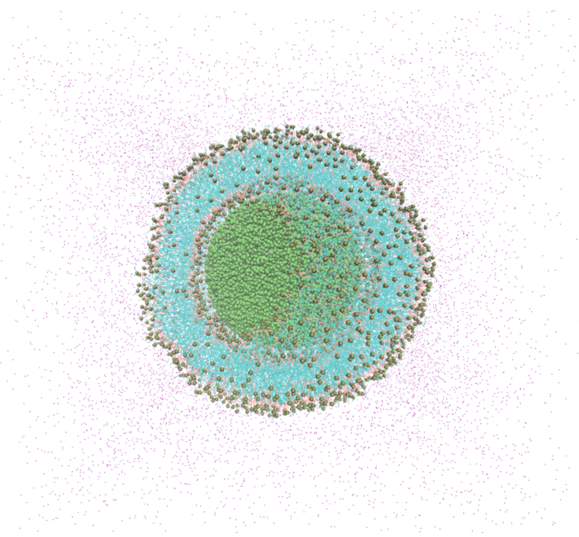

(g) 2.2μs

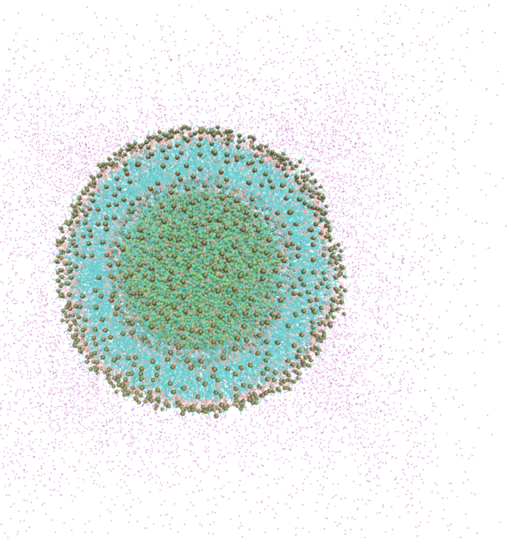

(h) 2.3 μs

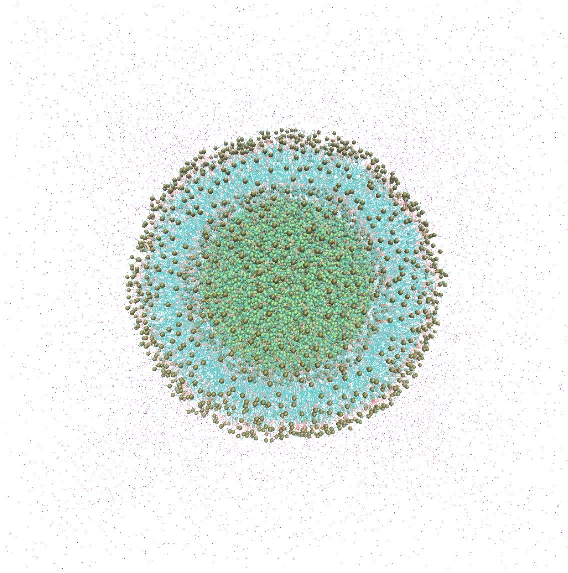

(I) 4.3 μs

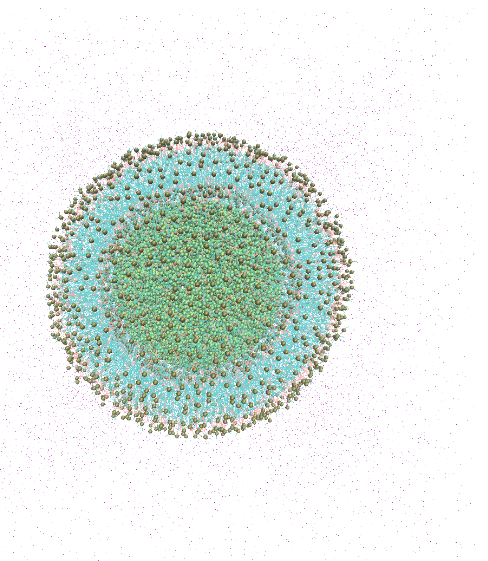

(J) 6.3 μs

Figure S2: MD simulation snapshots capturing the self-assembly driven equilibration of the NPSLBL system (system B or the system where the P5 beads constitute the NP of the NPSLBL and the lipids are the POPC molecules). The snapshot showing the initial configuration is provided in part (a) while the snapshots quantifying the progress of the simulation are shown in parts (b-h). Also, the snapshots from (a-e) represent the simulations conducted at 310 K, while

those from (f-h) represent the simulations conducted at 340 K. In (b) to (h), only 1/10th water are displayed for a clearer view. Below each snapshot, we provide the corresponding value of the time at which the snapshot has been taken. In the different subfigures, the following color codes have been used: green: NP; light green: hydrophobic tails of POPC molecules; bronze: hydrophilic heads of POPC molecules; purple: water. Part of the figure was reproduced from our previous paper.[2]

S2. MD simulations for the self-assembly of the PSSLBL

For the PSSLBL, the support substrate comprised of Nda[1] beads which are randomly distributed in a rectangle space of 20nm*20nm*1.6nm. The density of the Nda beads is same as the support substrate used in NPSLBL. This initial structure was obtained by using packmol[3] and we name system as *system C*. The self-assembly of *system C* was shown in figure S3.

Similar to NPSLBL, we substitute the Nda beads in *system C* with P5 beads and name the new system as *system D.* To save simulation time, we consider only 1250 POPC molecules randomly distributed above the substrate in the initial structure for *system D*. The self-assembly of *system D* was shown in figure S4.

The simulations are started from the initial structure shown in Fig. S3(a) and Fig. S4(a). The NAMD software package[4] was used to run the Simulations with the Martini force field.[1] Periodic boundary conditions were employed along with the NPT thermostat. The pressure was set to 1 bar, and simulation time step was set to 40fs. In addition, the temperature was set to 310K. The lipids quickly arranged and form a LBL above the substrate, becoming a PSSLBL within 8ns, and the PSSLBL is equilibrated for another 10ns. At equilibrium, there are 730 lipids at the inner leaflet and 770 lipids at the outer leaflet of the PSSLBL *for system C*.

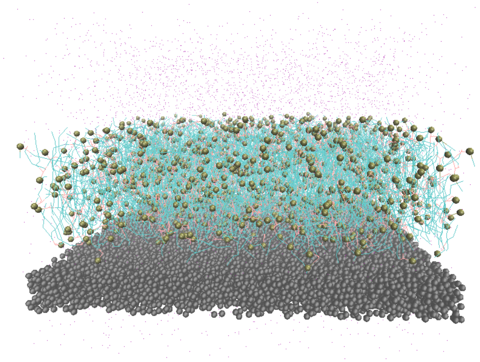

(a) 0 *ps*

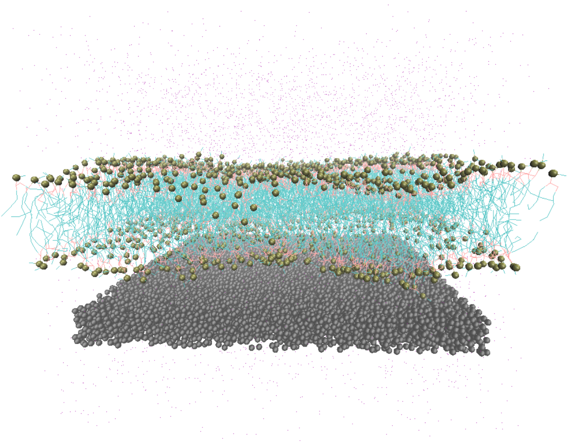

(b) 4 *ns*

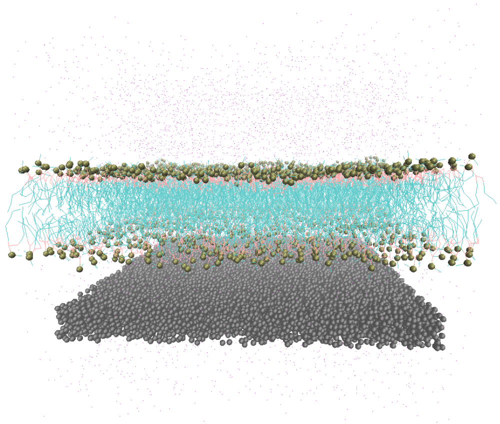

(c) *8ns*

Figure S3: MD simulation snapshots capturing the self-assembly driven equilibration of the PSSLBL system (system C or the system where the Nda beads constitute the NP of the NPSLBL and the lipids are the POPC molecules). Below each snapshot, we provide the corresponding simulation step at which the snapshot has been taken. The following color codes have been used:

black: support substrate; light green: hydrophobic tails of POPC molecules; bronze: hydrophilic heads of POPC molecules, purple: water.

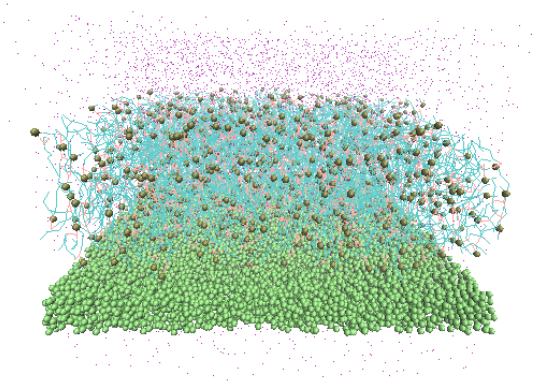

(a) 0 *ps*

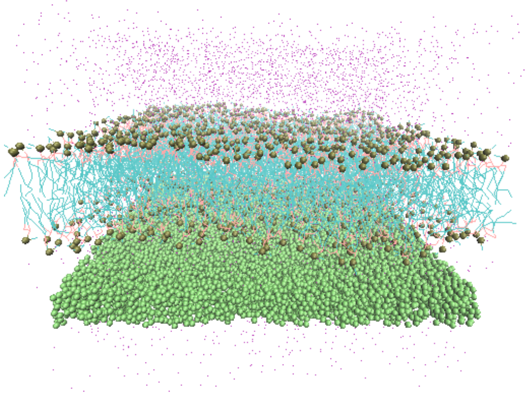

(b) 4 *ns*

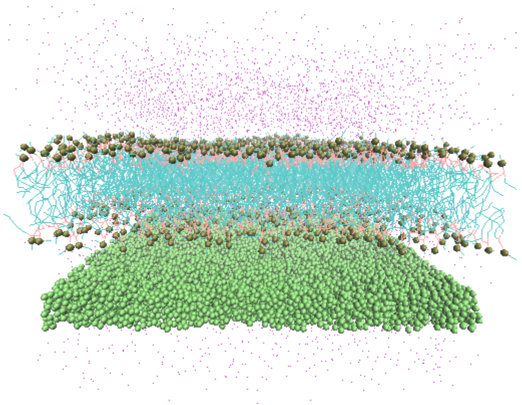

(c) *8ns*

Figure S4: MD simulation snapshots capturing the self-assembly driven equilibration of the PSSLBL system (system C or the system where the Nda beads constitute the NP of the NPSLBL and the lipids are the POPC molecules). Below each snapshot, we provide the corresponding simulation step at which the snapshot has been taken. The following color codes have been used:

green: support substrate; light green: hydrophobic tails of POPC molecules; bronze: hydrophilic heads of POPC molecules, purple: water.

S3. Convergence check of the simulations

To check the convergence of the simulation, we plot the thickness (*H*) and the area per lipid (*U*) of the LBL of *system C* as a function of time after 8ns in Fig. S5. From the figures, it is reasonable to believe the system has reached equilibrium. In addition, to check the convergence of the umbrella sampling, we show the PMF of the lipid flip and flop for systems A, B, C, and D at three different time (100ns, 200ns, 300ns) in figure S6. We can see that the PMF converged after 200ns. Furthermore, we also checked the overlapping of the umbrella windows (charcaterizing the flop and flip of the lipid molecule for systems A, B, C, and D) in figure S7 by showing the configuration histograms between 7 neighboring windows.

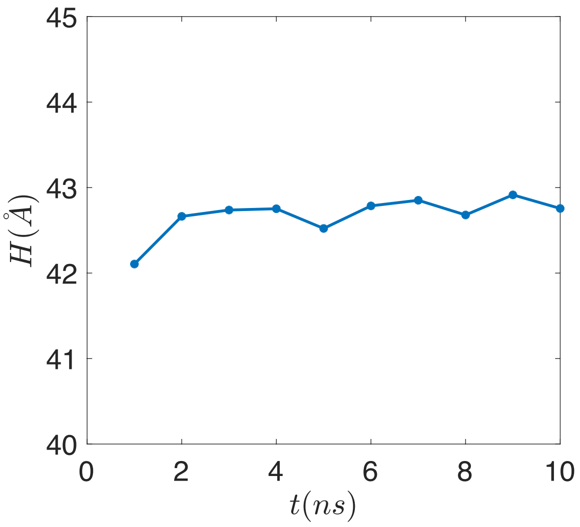

(a)

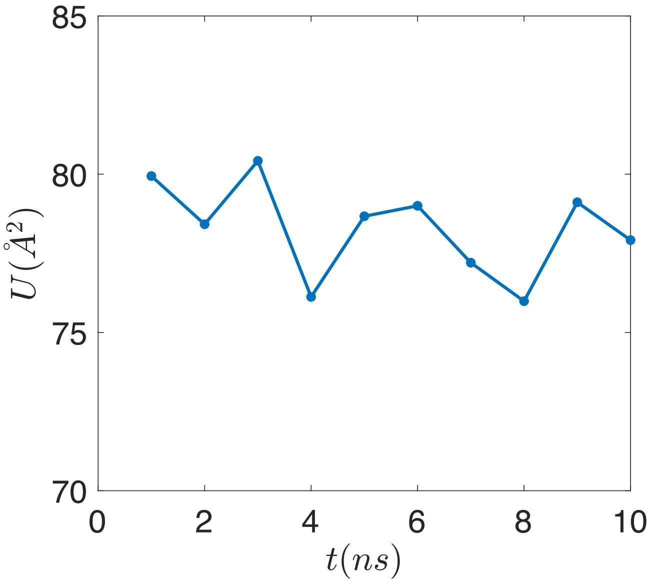

(b)

Figure S5: (a) thickness (*H*) and (b) area per lipid (*U*) of the LBL of *system C* as a function of time after 8ns. The horizonal axis time *t* is the real time minus 8ns.

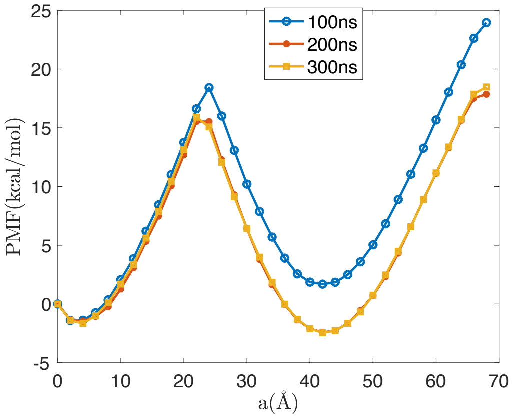
100ns
200ns
300ns
PMF(kcal/mol)
a(Å)

(a)

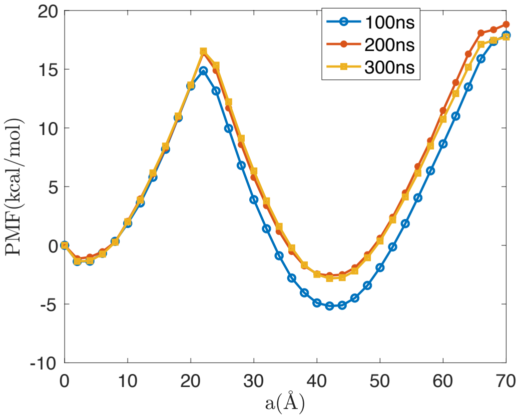
100ns
200ns
300ns
PMF(kcal/mol)
a(Å)

(b)

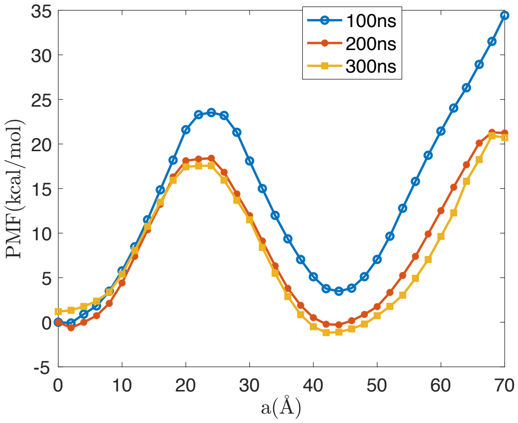
100ns
200ns
300ns
PMF(kcal/mol)
a(Å)

(c)

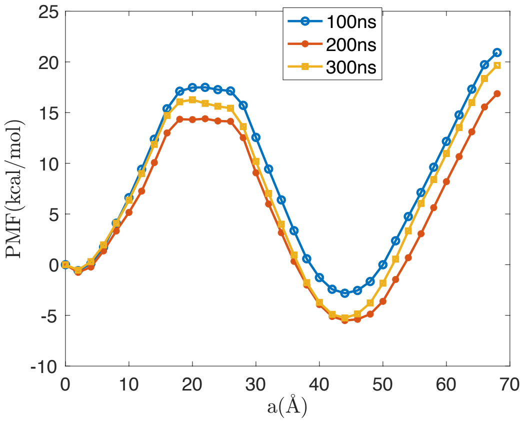
100ns
200ns
300ns
PMF(kcal/mol)
a(Å)

(d)

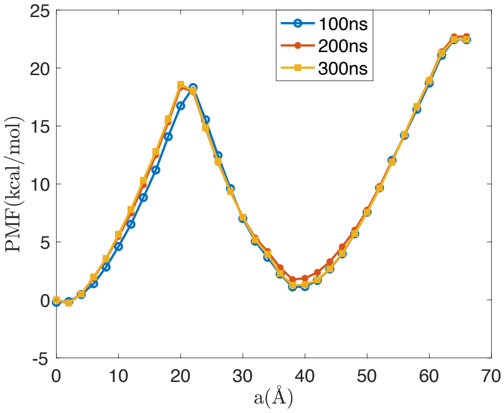
100ns
200ns
300ns
PMF(kcal/mol)
a(Å)

(e)

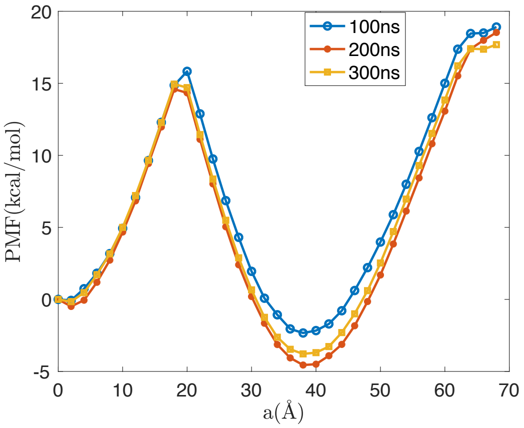
100ns
200ns
300ns
PMF(kcal/mol)
a(Å)

(f)

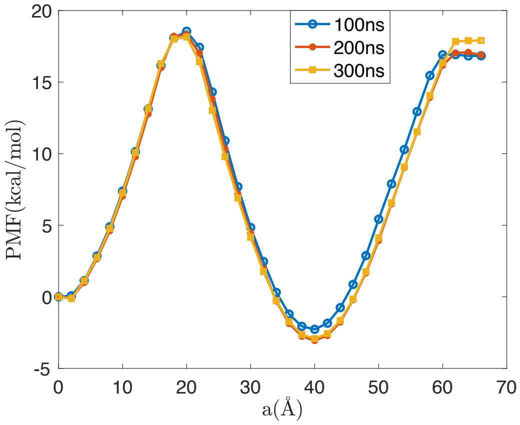

(g)

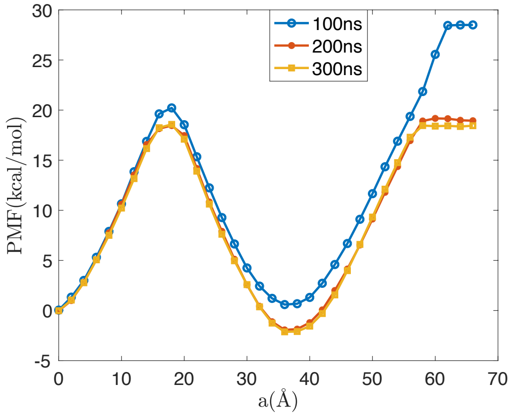

(h)

Figure S6: PMFs at three different times (100ns, 200ns, 300ns) for (a) flop in *system A,* (b) flip in *system A,* (c) flop in *system C,* (d) flip in *system C,* (e) flop in *system B,* (f) flip in *system B,* (g) flop in *system D,* and (h) flip in *system D.*

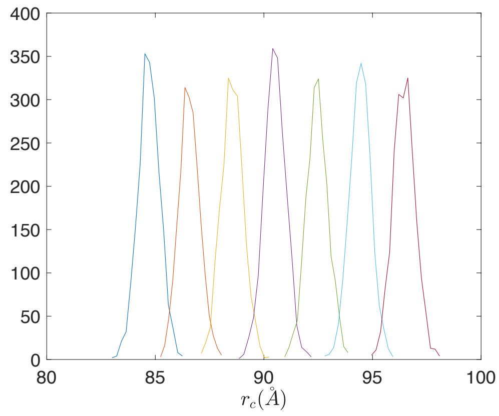
0 50 100 150 200 250 300 350 400
80 85 90 95 100
$r_c(\AA)$

(a)

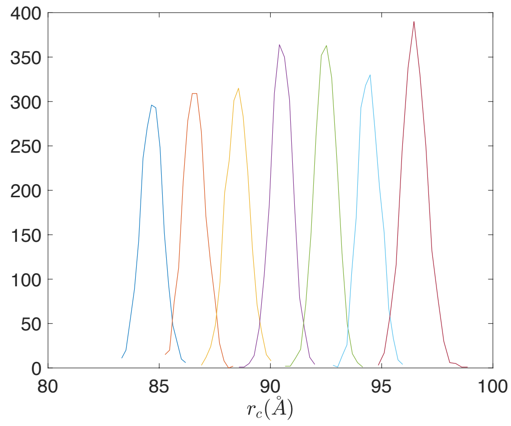
0 50 100 150 200 250 300 350 400
80 85 90 95 100
$r_c(\AA)$

(b)

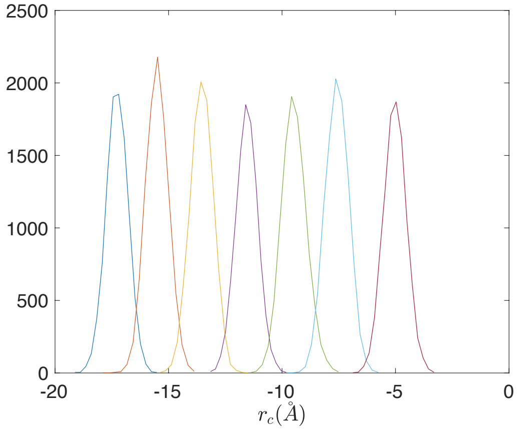
0 500 1000 1500 2000 2500
-20 -15 -10 -5 0
$r_c(\AA)$

(c)

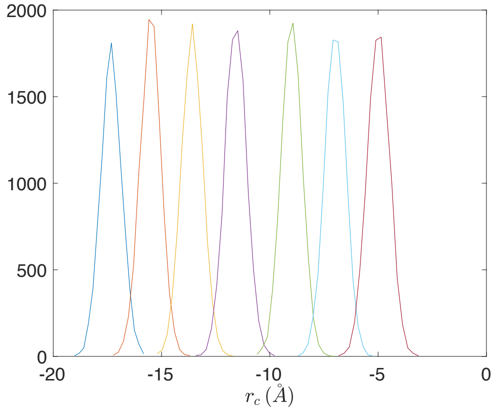
0 500 1000 1500 2000
-20 -15 -10 -5 0
$r_c\,(\AA)$

(d)

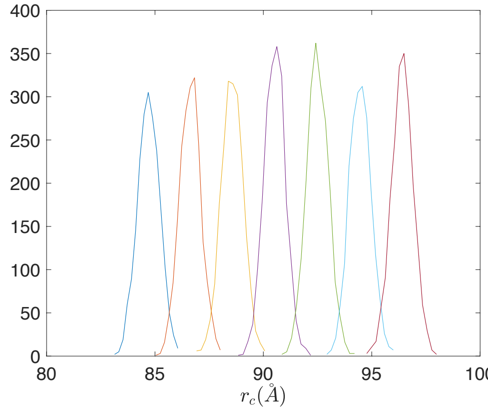
0 50 100 150 200 250 300 350 400
80 85 90 95 100
$r_c(\AA)$

(e)

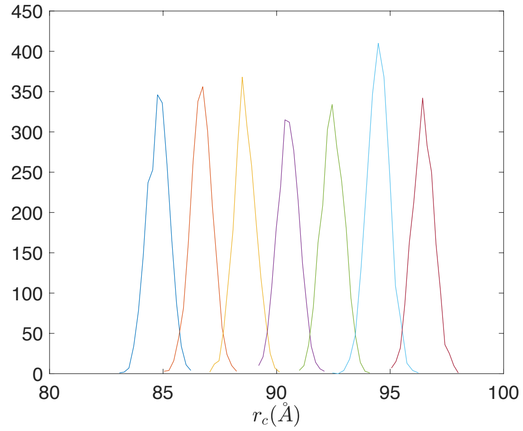
0 50 100 150 200 250 300 350 400 450
80 85 90 95 100
$r_c(\AA)$

(f)

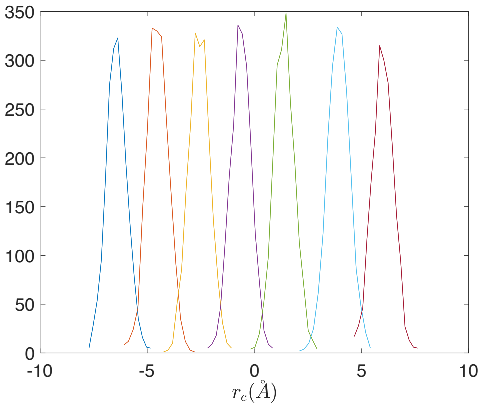

(g)

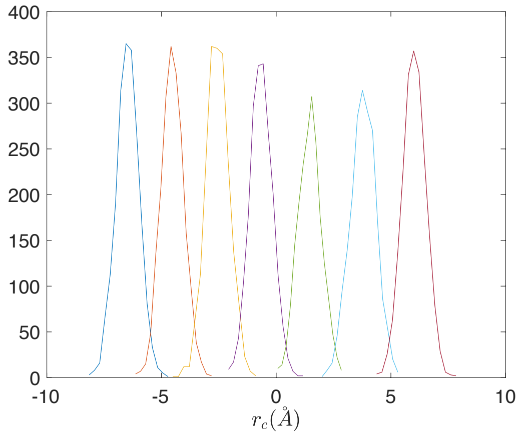

(h)

Figure S7: 7 adjacent umbrella windows (demosntrating their overlaps) for (a) flop in *system A,* (b) flip in *system A,* (c) flop in *system C,* (d) flip in *system C,* (e) flop in *system B,* (f) flip in *system B,* (g) flop in *system D,* (h) flip in *system D.*

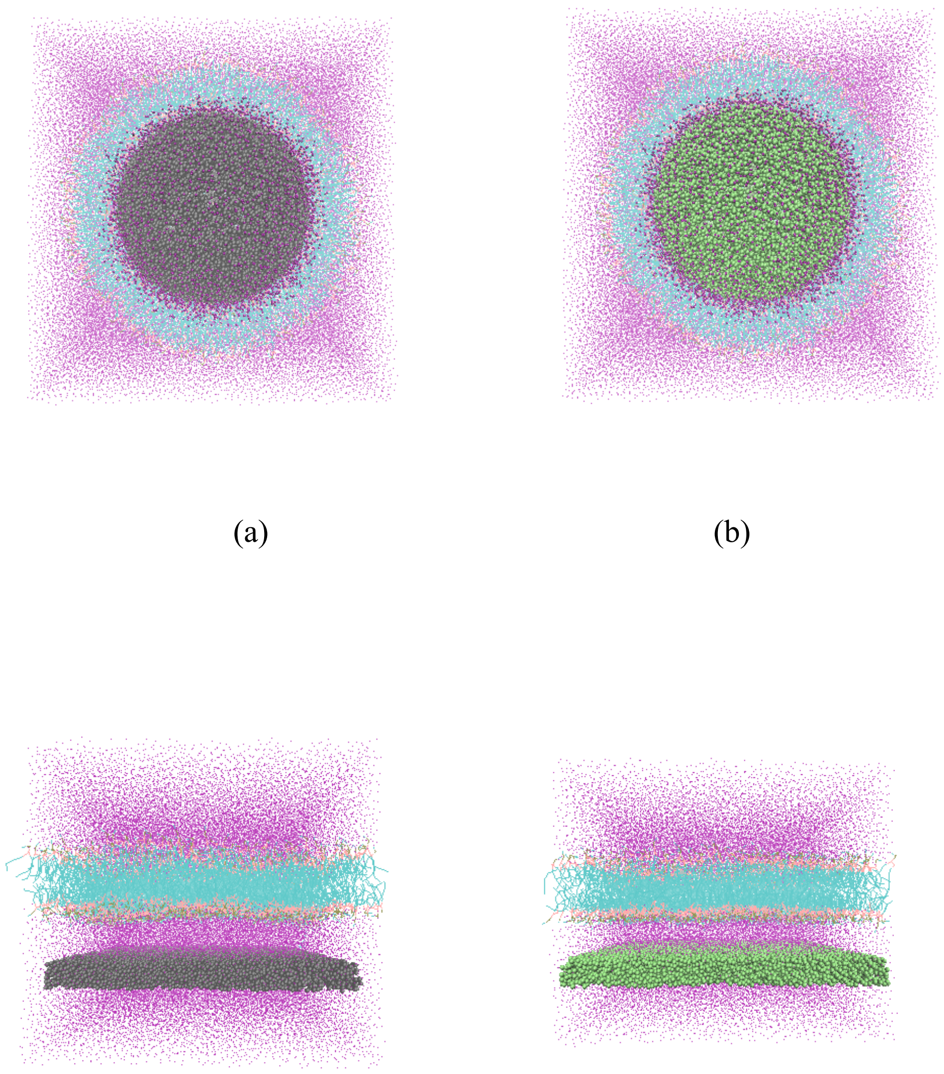

(a) (b) (c) (d)

Figure S8: snapshots show the existence of confined water between NP and LBL. (a) *System A* (b) *System B* (c) *System C* and (d) *System D*. The following color codes have been used: black: Nda NP/substrate; green: P5 NP/substrate; light green: hydrophobic tails of POPC molecules; orange: hydrophilic heads of POPC molecules, purple: water.

S4. Supporting Files

The psf, pdb and dcd for the equilibration states for the simulation are provided at

https://drive.google.com/drive/folders/17KDiUsWjbjsdKkBUi3a4hWoCxo9R_FnB?usp=sharing

The files are named with A, B, C, D for *system A, B, C and D* respectively.

Reference:

1. Marrink, S. J.; Risselada, H. J.; Yefimov, S.; Tieleman, D. P.; de Vries, A. H. The MARTINI Force Field: Coarse Grained Model for Biomolecular Simulations. *J. Phys. Chem. B* **2007**, *111*, 7812-7824.
2. Jing, H.; Wang, Y.; Desai, P. R.; Ramamurthi, K.; Das, S. Formation and Properties of Self-Assembled Nanoparticle-Supported Lipid Bilayer Probed Through Molecular Dynamics Simulations. *Langmuir* **2020**, 36, 5524-5533.
3. Martínez, L.; Andrade, R.; Birgin, E. G.; Martínez, J. M. PACKMOL: A Package for Building Initial Configurations for Molecular Dynamics Simulations. *J. Comput. Chem.* **2009**, *30*, 2157-2164.
4. Phillips, J. C.; Braun, R.; Wang, W.; Gumbart, J.; Tajkhorshid, E.; Villa, E.; Chipot, C.; Skeel, R. D.; Kale, L.; Schulten, K. Scalable Molecular Dynamics with NAMD. *J. Comput. Chem.* **2005**, *26*, 1781-1802.
